# Comprehensive global genome dynamics of *Chlamydia trachomatis* show ancient diversification followed by contemporary mixing and recent lineage expansion

**DOI:** 10.1101/gr.212647.116

**Published:** 2017-07

**Authors:** James Hadfield, Simon R. Harris, Helena M.B. Seth-Smith, Surendra Parmar, Patiyan Andersson, Philip M. Giffard, Julius Schachter, Jeanne Moncada, Louise Ellison, María Lucía Gallo Vaulet, Marcelo Rodríguez Fermepin, Frans Radebe, Suyapa Mendoza, Sander Ouburg, Servaas A. Morré, Konrad Sachse, Mirja Puolakkainen, Suvi J. Korhonen, Chris Sonnex, Rebecca Wiggins, Hamid Jalal, Tamara Brunelli, Patrizia Casprini, Rachel Pitt, Cathy Ison, Alevtina Savicheva, Elena Shipitsyna, Ronza Hadad, Laszlo Kari, Matthew J. Burton, David Mabey, Anthony W. Solomon, David Lewis, Peter Marsh, Magnus Unemo, Ian N. Clarke, Julian Parkhill, Nicholas R. Thomson

**Affiliations:** 1 Pathogen Genomics, The Wellcome Trust Sanger Institute, Wellcome Trust Genome Campus, Hinxton, Cambridge CB10 1SA, United Kingdom;; 2 Public Health England, Public Health Laboratory Cambridge, Addenbrooke's Hospital, Cambridge CB2 0QW, United Kingdom;; 3 Menzies School of Health Research, Darwin, Northern Territory 0810, Australia;; 4 School of Psychological and Clinical Sciences, Charles Darwin University, Darwin 0909, Australia;; 5 Department of Laboratory Medicine, University of California at San Francisco, San Francisco, California 94110, USA;; 6 Universidad de Buenos Aires, Facultad de Farmacia y Bioquímica, Departamento de Bioquímica Clínica, Microbiología Clínica, Buenos Aires C1113AAD, Argentina;; 7 Centre for HIV and Sexually Transmitted Infections, National Institute for Communicable Diseases, National Health Laboratory Service, 2192 Johannesburg, South Africa;; 8 Jefe Laboratorio de ITS, Laboratorio Nacional de Vigilancia, FM1100, Honduras;; 9 Department of Medical Microbiology and Infection Control, Laboratory of Immunogenetics, VU University Medical Center, 1081 HZ Amsterdam, The Netherlands;; 10 Department of Genetics and Cell Biology, Institute of Public Health Genomics, School for Oncology & Developmental Biology (GROW), Faculty of Health, Medicine and Life Sciences, University of Maastricht, 6229 ER Maastricht, The Netherlands;; 11 Institute of Molecular Pathogenesis, Friedrich-Loeffler-Institut (Federal Research Institute for Animal Health), 07743 Jena, Germany;; 12 Department of Virology, University of Helsinki and Helsinki University Hospital, University of Helsinki, 00014 Helsinki, Finland;; 13 Department of Biology, University of York, York CB2 2QQ, United Kingdom;; 14 Clinical Chemistry and Microbiology Laboratory, Santo Stefano Hospital, ASL4, 59100 Prato, Italy;; 15 Sexually Transmitted Bacteria Reference Unit, Microbiological Services, Public Health England, London NW9 5HT, United Kingdom;; 16 Laboratory of Microbiology, D.O. Ott Research Institute of Obstetrics and Gynecology, St. Petersburg, Russia 199034;; 17 WHO Collaborating Centre for Gonorrhoea and other STIs, Faculty of Medicine and Health, Örebro University Hospital, SE-701 85 Örebro, Sweden;; 18 Laboratory of Intracellular Parasites, Rocky Mountain Laboratories, National Institute of Allergy and Infectious Diseases, National Institutes of Health, Hamilton, Montana 59840, USA;; 19 Clinical Research Department, Faculty of Infectious and Tropical Diseases, London School of Hygiene & Tropical Medicine, London WC1E 7HT, United Kingdom;; 20 Centre for Infectious Diseases and Microbiology and Marie Bashir Institute for Infectious Diseases and Biosecurity, Westmead Clinical School, University of Sydney, Sydney 2192, Australia;; 21 Public Health England, Public Health Laboratory Southampton, Southampton General Hospital, Southampton SO16 6YD, United Kingdom;; 22 Molecular Microbiology Group, University Medical School, Southampton General Hospital, Southampton SO16 6YD, United Kingdom;; 23 Department of Pathogen Molecular Biology, The London School of Hygiene and Tropical Medicine, London WC1 7HT, United Kingdom

## Abstract

*Chlamydia trachomatis* is the world's most prevalent bacterial sexually transmitted infection and leading infectious cause of blindness, yet it is one of the least understood human pathogens, in part due to the difficulties of in vitro culturing and the lack of available tools for genetic manipulation. Genome sequencing has reinvigorated this field, shedding light on the contemporary history of this pathogen. Here, we analyze 563 full genomes, 455 of which are novel, to show that the history of the species comprises two phases, and conclude that the currently circulating lineages are the result of evolution in different genomic ecotypes. Temporal analysis indicates these lineages have recently expanded in the space of thousands of years, rather than the millions of years as previously thought, a finding that dramatically changes our understanding of this pathogen's history. Finally, at a time when almost every pathogen is becoming increasingly resistant to antimicrobials, we show that there is no evidence of circulating genomic resistance in *C. trachomatis*.

*Chlamydia trachomatis* infections, including their severe sequelae, are major public health concerns globally, resulting in significant morbidity and health care costs (Owusu-Edusei et al. 2013). *C. trachomatis* has been estimated to be the most common bacterial sexually transmitted infection (STI) worldwide, with 131 million cases among adults annually (World Health Organisation 2014; Newman et al. 2015). Furthermore *C. trachomatis* is the etiological agent of the severe ocular disease trachoma, with an estimated 232 million people at risk of blindness worldwide (World Health Organisation 2014).

Our clinical and epidemiological understanding of *C. trachomatis* is primarily based upon typing of the *ompA* gene: genotypes A, B, Ba, and C associate with trachoma (ocular lineage); D, E, F, G, H, I, Ia, J, and K with urogenital infection (lineages T1 and T2); and L1, L2, L2b, L2c, and L3 with the more invasive lymphogranuloma venereum (LGV) lineage (Harris et al. 2012). Biologically, despite the large number of cases and the potential severity of infections, *C. trachomatis* is poorly understood due to its obligate intracellular lifestyle, the difficulties in growing strains in vitro, and, until recently, a lack of genetic tools for manipulation and transformation (Wang et al. 2011; Song et al. 2013).

Genomics has provided an alternative route to understanding this disease and has shown that *C. trachomatis* has evolved through reductive evolution, likely from nonpathogenic ancestors that became specialized to humans (Stephens et al. 1998; Borges et al. 2012). *C. trachomatis* comprises a ∼1-Mb chromosome and ∼7-kb plasmid (Stephens et al. 1998). The species phylogeny consists of four deeply branching lineages that are strongly, but not exclusively, associated with site of infection (Joseph et al. 2011; Borges et al. 2012; Harris et al. 2012; Andersson et al. 2016).

Homologous recombination, pervasive across the bacterial kingdom (Feil et al. 2001), was initially thought to be uncommon in *C. trachomatis* owing to its intracellular niche (Fraser et al. 2007; Nunes et al. 2007). However, recent whole-genome analyses have shown that recombination is widespread across multiple species of the *Chlamydiaceae* with the ratio of diversity introduced by recombination and mutation (r/m) estimated at between 0.56 and 3.19 (r/m 0.71–1.11 for *C. trachomatis*), and certain regions of the genome, including the clinically and epidemiologically relevant *ompA*, exhibit elevated levels of fixed, and therefore detectable, recombinations (Brunelle and Sensabaugh 2005; Klint et al. 2007; Joseph et al. 2011, 2012; Harris et al. 2012; Read et al. 2013). This is backed up experimentally, with in vitro coinfections of several *C. trachomatis* strains leading to extensive homologous recombination (Jeffrey et al. 2013), and supported by epidemiological estimates of mixed infections at between 2% and 13% (DeMars et al. 2007). These studies indicate that recombination is possibly the more important mechanism generating diversity in this species.

Antimicrobial resistance is currently recognized as one of the most critical threats to human health. Many bacterial species are becoming fully resistant to all frontline drugs, including sexually transmitted bacteria such as *Neisseria gonorrhoeae* and *Mycoplasma genitalium,* which commonly coinfect with *C. trachomatis* (Unemo and Shafer 2011). Despite treatment failures of *C. trachomatis* infections with recommended therapeutic antimicrobials being reported (Hogan et al. 2004), isolated reports of genomically conferred resistance (Misyurina et al. 2004; Jiang et al. 2015), and the ease with which resistance can be selected for in vitro by exposure to subinhibitory antimicrobial concentrations (Sandoz and Rockey 2010), little is known about the prevalence, if any, of circulating resistance traits.

This study was motivated by the fact that our current genomic view of *C. trachomatis* is based on whole-genome data sets of at most 59 genomes, limited largely by sample collection technology and sequencing cost. There remain many unanswered questions, including the age of *C. trachomatis*, the rate of evolution, and the evolutionary differences between and within the four deep-branching lineages. The rate of single-nucleotide polymorphism (SNP) accumulation is particularly important as estimating it would allow us to better understand the dynamics of infection transmission. In addition, there is a critical need to establish whether antimicrobial resistance has begun to circulate in clinical isolates. This study sought to elucidate, in a detailed manner, the evolution of the *C. trachomatis* species by sequencing a worldwide collection of hundreds of current and historical clinical strains, increasing the sample size studied for this pathogen by nearly an order of magnitude. This study forms the most comprehensive and robust analysis of *C. trachomatis* genomics yet undertaken, includes cultured isolates as well as those sequenced directly from primary clinical specimens, and provides clear insights into the evolutionary history and dynamics, which may have implications for diagnosis, epidemiology, and human public health worldwide.

## Results

We present the largest and broadest genomic and evolutionary study of *C. trachomatis* published to date, comprising 563 full genomes of isolates collected between 1957 and 2012, 455 of which are reported here for the first time (Supplemental Table S1). We strove to obtain samples without, or with minimal, laboratory passage in order to assemble a set of isolates representative of clinical and in vivo evolution. Recent work examining the impact of in vitro culture has shown that pseudogenization is directly associated with prolonged laboratory passage, emphasizing the importance of culture-free techniques to fully understand natural patterns of evolution (Bonner et al. 2015). This entailed the use of culture-free DNA extraction methods from primary diagnostic swab samples and direct-from-sample sequencing strategies developed by us and others to complement the samples grown by minimal culture (Seth-Smith et al. 2013a; Christiansen et al. 2014). We obtained 76 genomes using multiple displacement amplification (MDA), 103 with additional immunomagnetic separation (IMS-MDA), and 77 samples by SureSelect target enrichment, providing 170 novel genomes obtained without the use of culture. Our sampling included clinical samples from a diverse cross-section of countries (*n* = 21) in Europe, North America, South America, Africa, Asia, and Australia. All known genotypes are represented, including isolates that do not fit the generally understood linkage between genotype and site of infection. Historical archived samples dating to the 1950s are also included. Together, this data set provides a unique basis to answer subtle, but fundamental, questions about the evolutionary flux in this important human pathogen.

### The species consists of four deep-branching lineages

**Figure 1.**
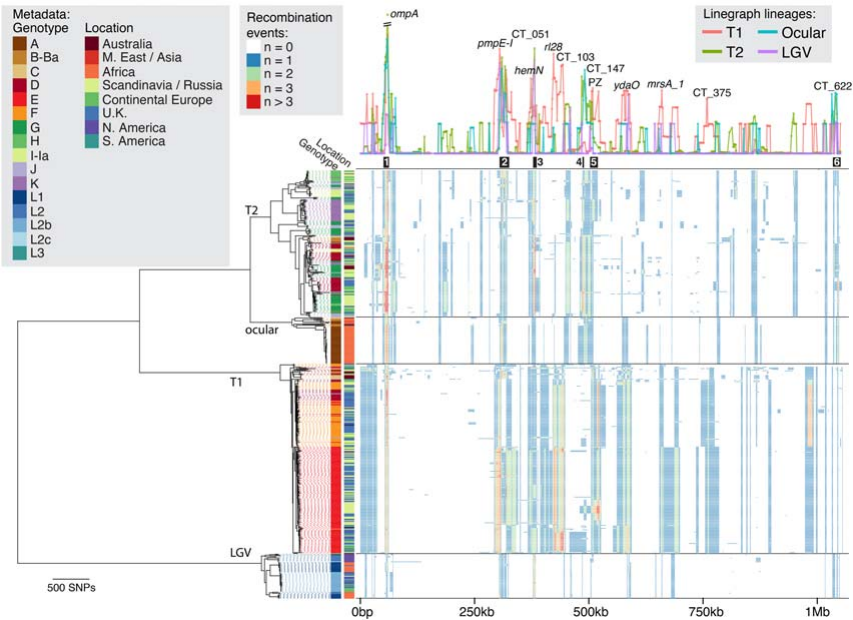
Global phylogeny and recombination landscape of 563 *C. trachomatis* genomes. The phylogeny (*left*), with associated genotype and geographical data, is displayed alongside the linearized chromosome (*right*). Lineage labels are as per previous publications. Line graph (*right*, *top*) shows the number of recombination events affecting individual genes and are colored according to lineage. Black blocks *below* this graph show previously identified “hotspots” of recombination (Harris et al. 2012). Colored blocks (*right*, *bottom*) indicate inferred recombination events each affecting selected taxa and genomic location, with color indicating the number of inferred events. Gene annotations correspond to strain D/UW3. (PZ) Plasticity zone.

The global phylogeny of all samples sequenced thus far (Fig. 1) confirms that the species consists of four deep-branching monophyletic lineages that generally associate with tropism—two urogenital lineages, T1 (*n* = 250) and T2 (*n* = 193); an ocular lineage (*n* = 61); and a LGV lineage (*n* = 59)—consistent with previous reports (Harris et al. 2012; Joseph et al. 2012; Nunes and Gomes 2014). Plasmid inheritance was almost entirely vertical at this resolution except for three cases where plasmid replacement from other lineages has occurred (Supplemental Fig. S1); in addition, the plasmid phylogeny associated with lineage T2 is paraphyletic due to an apparent recombination event (Supplemental Fig. S2).

**Figure 2.**
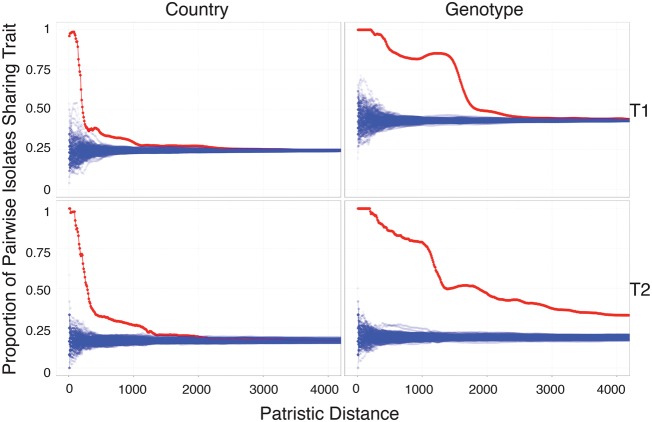
Proportion of pairwise isolates sharing a given trait (country or genotype) as a function of genomic divergence for the well-sampled urogenital lineages T1 (*top*) and T2 (*bottom*). (*Top*, *left*) For instance any pair of isolates less than approximately 400 mutations apart contain the same trait (country of isolation) in 50% of cases. (Red) Observed data; (blue) 100 permutations; (*left*) country of isolation; (*right*) genotype.

Geographical location is significantly associated with phylogenetic distance in the urogenital lineages (Fig. 2). This information allows us to say that isolates fewer than 210 (T1) or 280 (T2) SNPs apart are more than 50% likely to come from the same country, whereas for isolates to have a 50% probability of sharing the same genotype, the distances are much larger: 1850 SNPs and 1470 SNPs for lineages T1 and T2, respectively. Genotype, as expected, is also significantly correlated with evolutionary distance (Fig. 2) and, furthermore, is strongly associated with lineage (Table 1). We see no association between the gender of the patient and evolutionary separation of strain. Limited geographical sampling in the ocular and LGV lineages prohibits us from drawing definitive conclusions, although we note that all LGV isolates were sampled from males due to current transmission patterns of this clade. Culture-free isolates show phylogenetic clustering most likely due to the nonrandom nature of sample collection and processing. Importantly, we see both cultured and culture-free extraction methods providing samples that locate throughout the tree, indicating that there is no sizeable diversity missed by culture methods alone.

**Table 1.**
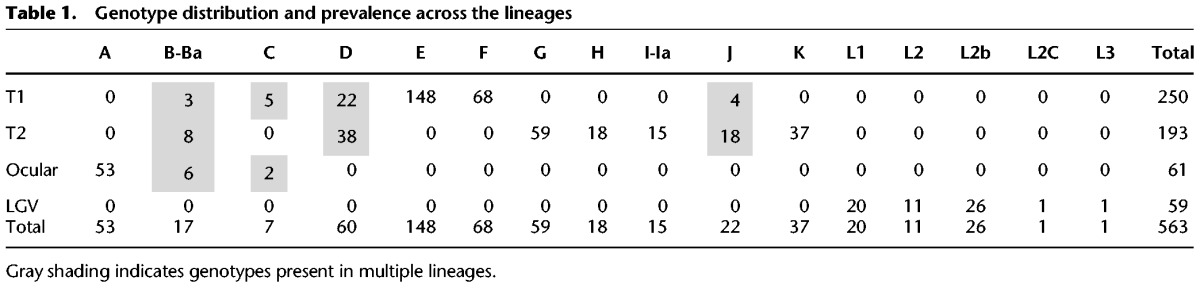
Genotype distribution and prevalence across the lineages

Of the 51 countries known or suspected to be endemic for trachoma (World Health Organisation 2014), we have samples from only six. This limited range reflects the fact that most control programs do not routinely collect samples, so our sampling is largely limited to the areas that have active research projects; therefore, we are limited to analyzing small pockets of genetic variation for this disease. A recent study investigating trachoma in the Aboriginal population of Australia showed that isolates with genotypes B, Ba, and C were found outside the ocular lineage and were associated with ocular disease (Andersson et al. 2016), as indicated in Supplemental Figure S3. Within this study, we find isolates with the same genomic backbone, including genotype, as those from Australia in urogenital samples from Europe (Supplemental Fig. S3), indicating that these strains are not restricted to Australia.

Previous studies with relatively small sample sizes have allowed identification of the broad differences between lineages but have not been able to define evolutionary characteristics within the lineages themselves. The phylogeny shown in Figure 1 elucidates the contrasting appearance of the two urogenital lineages. T1, in comparison with T2, contains fewer vertically inherited SNPs as evidenced by its “flatter” appearance. T1, the most sampled lineage in this study, contains a monophyletic expansion consisting almost entirely of genotype E isolates (*n* = 148/149) (Table 1). Genotype E is the most epidemiologically successful genotype worldwide and is entirely contained within this expansion, indicating a relatively recent emergence where recombination has not disrupted the *ompA* gene, yet one that has spread worldwide. This contrasts with previous studies showing that *ompA* has the highest rates of observed recombination, which results in frequent genotype switching across the species (Harris et al. 2012).

A comparison of phylogenies with and without predicted recombination events (Supplemental Fig. S4) left the overall structure of four lineages firmly intact, indicating that recombination events have not introduced sufficient homoplasy to disrupt this evolutionary signal. Recombinations have, however, resulted in extensive intra-lineage changes in topology, a discordance that indicates that recombination must be taken into account when trying to understand the evolution of the species on detailed level.

### Lineage-specific patterns of recombination

The evolutionary, clinical, and epidemiological significance of recombination in *C. trachomatis* has recently become apparent, and we have investigated these dynamics in much greater detail than previously possible. By examining all 563 genomes in the present study, we detected 1116 putative recombination blocks (Fig. 1, heatmap) spanning an average of 7.5 kb per recombination (95% CI: 7.0–8.0 kb) and covering an average of 246 kb per isolate (23% of the genome) with an average r/m of 0.31. Hotspots of recombination were originally observed around the *ompA* locus (Gomes et al. 2006) and subsequently detected to be nonuniformly distributed across the genome (Harris et al. 2012). It is clear that with deeper sampling, the recombination landscape is more dynamic than would be expected from a small number of hotspots (Fig. 1). The six previously identified regions of higher than average recombination (Harris et al. 2012) are identified here, including region 1 (around *ompA*), region 2 (including *pmpE-I*), region 3 (around CT051 and *hemN*), and region 6 (around CT622). We find that regions 4 and 5 are bridged into one continuous region of increased recombination that encompasses the plasticity zone (PZ) (Fig. 1). While these regions show increased rates of recombination in all lineages, the majority of recombination is specific to a lineage or a clade within a lineage, such as between regions 3 and 4 (spanning *rl28* to CT103) that has markedly increased recombination present only in lineage T1. Indeed, the overall effect of recombination varies nearly 10-fold between lineages, from r/m = 0.37 (ocular) to 3.11 (T1) (Table 2). The two urogenital lineages also experience markedly differing amounts of recombination (r/m 1.23–3.11), a result that leads to dramatic differences in how recombination shapes their respective phylogenies (Table 1; Supplemental Fig. S4), further suggesting that T1 is a more recent lineage that has experienced expansion driven by recombination. These observations are only made possible by larger and more representative sampling and confirm that our previous views were too simplistic. Variation in recombination across a genome may be mechanistic, selective, or stochastic. There is no evidence for mechanistic differences (Gomes et al. 2006), and the consistency of locations of recombination across samples refutes a stochastic process, leading us to conclude that we are seeing the signatures of selective pressures on multiple levels: the species as a whole, a lineage, or an expansion within a lineage.

**Table 2.**
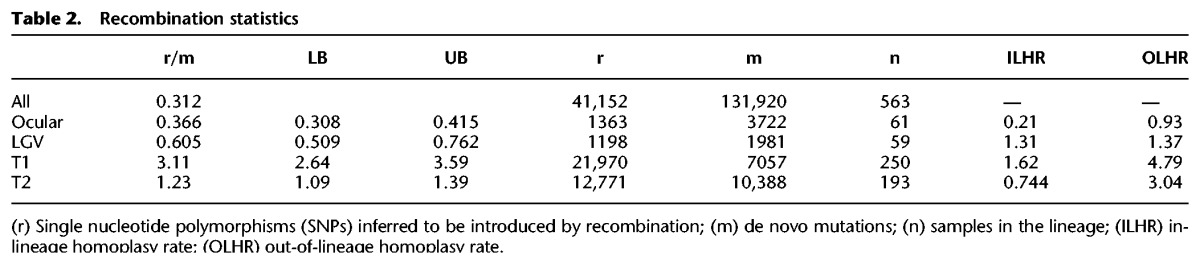
Recombination statistics

**Figure 3.**
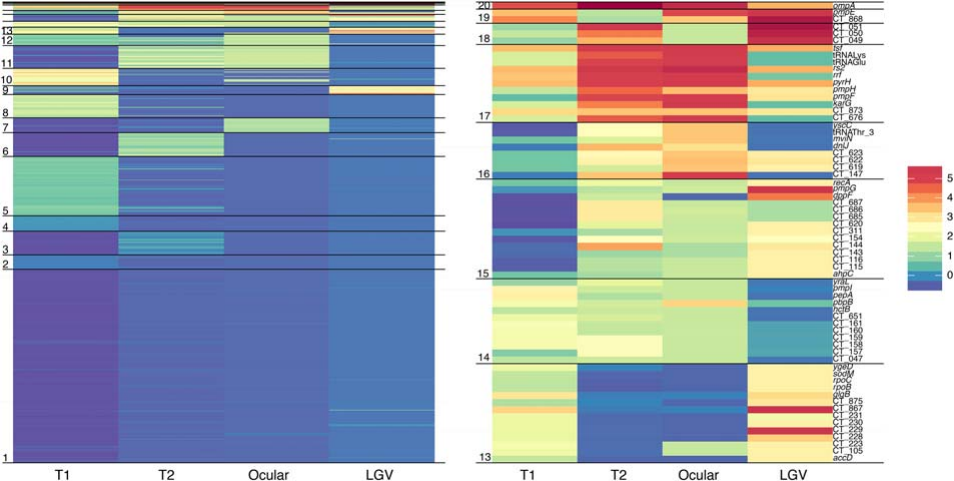
Clustering of genes by recombination frequency in each lineage reveals lineage-specific profiles. Each horizontal line represents a gene with colors corresponding to standard deviations from the clade-specific mean. (*Right*) Expansion of the seven most actively recombining clusters. Clusters do not necessarily represent order of genes along the genome.

These lineage-specific evolutionary traits provide for the first time important clues as to the roles of genes in different host environments. To define the genes in these recombination regions that are lineage specific, we clustered genes according to the number of observed recombination events per lineage. This clustering (Fig. 3; Supplemental Table S2) helps us to elucidate sets of genes under differing selective pressures in different lineages. For example, we found a set of 18 genes, including the inclusion membrane protein genes *incA* and *incB,* that appears to be under increased selective pressure in the LGV clade. *Inc* genes are known immune targets, and these results may indicate a selective host pressure specific to this lineage. As in vitro experiments often use LGV strains (owing to their ease of infection in cell culture), these data should be consulted to ensure that the genes of interest are not under differing dynamics in the LGV lineage alone.

### Evolutionary timeline for the emergence of *C. trachomatis*

**Figure 4.**
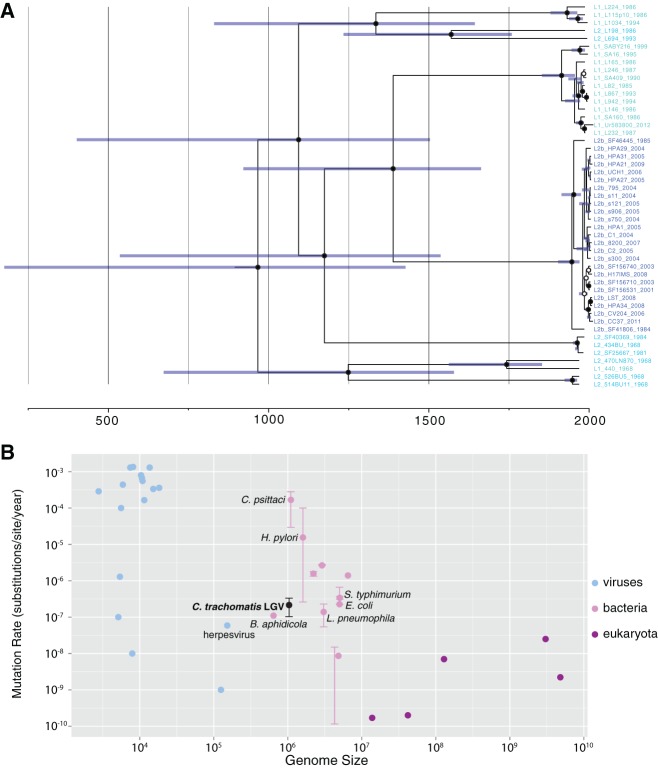
(*A*) Temporal analysis of the LGV clade indicates a most recent common ancestor (MRCA) between 200 CE and 1430 CE (95% highest posterior density [HPD]). Dates along the *x*-axis are in years (CE), and blue bars show the 95% posterior probability. Posterior probabilities of node positions are indicated by closed circles (*P* = 1) or open circles (*P* > 0.8). (*B*) The *Chlamydia trachomatis* LGV mutation rate is shown in the context of other viruses, bacteria and eukaryotes. Error bars differ per species according to methodology, but for the case of *C. trachomatis* represent 95% posterior probability. Data sources shown in Supplemental Table S3. *Buchnera aphidicola*, another intracellular bacterium, has a similar genome size and mutation rate.

The temporal history and mutation rate of *C. trachomatis* is poorly understood and has never been directly calculated. Root-to-tip analysis shows that the LGV clade contains significant temporal structure (*R*^2^ = 0.62, permuted *P* ≤ 0.001) (Supplemental Fig. S5). This clade contained a number of isolates dating to the 1960s, sequenced here from stocks archived in the 1960s. A Bayesian analysis of these isolates using BEAST (Drummond and Rambaut 2007) placed the most recent common ancestor (MRCA) of the LGV clade around 900 CE (common era) (95% highest posterior density [HPD] 200 CE–1430 CE) (Fig. 4A; Supplemental Fig. S5). These results are dramatically different to a previous estimate of 15 Myr ago (Joseph et al. 2012), which used estimates of the split between *C. trachomatis* and *C. pneumoniae*, rather than our historical sampling approach. Our data predict that the L2b isolates arose about 100 yr ago, which fits with epidemiological reports (Schachter and Moncada 2005).

Our estimated substitution rates for this lineage were 2.15 × 10^−7^ SNPs/site/year (95% HPD 1.03 × 10^−7^–3.33 × 10^−7^), i.e., 0.2 SNPs per genome per year, which is consistent with rates reported for other bacteria, including the intracellular *Buchnera aphidicola* (Fig. 4B). The recently published substitution rate for the closely related *Chlamydia psittaci* of 1.68 × 10^−4^ mutations/site/yr (∼175 SNPs/yr) (Read et al. 2013) is nearly three orders of magnitude faster than that reported here and, when applied to these data, would predict that all modern *C. trachomatis* strains share a common ancestor within the past century, which is not possible. Analysis of other lineages showed some correlation between age and root-to-tip distance; however, this was not significant and could be an artifact of population structure, unidentified recombination events, or the scarcity of remaining vertically inherited SNPs. Long-term laboratory passage results in very few adaptive mutations, indicating that this is not a major source of bias (Borges et al. 2013). While it is perhaps unwise to extend the results from the LGV lineage to other lineages, doing so would indicate that all lineages are expansions during a timescale measured in thousands or in tens of thousands of years rather than in millions.

### The origins of *ompA*

The *ompA* gene has strong relevance for clinical, epidemiological, and public health understanding, despite recombining frequently and at different rates compared with the rest of the genome. Our results clarify the usefulness and limitations of using *ompA* for epidemiology or phylogenetic inference. The majority of genotypes (12/16) are restricted to single lineages, with a further two (D, J) each found in both urogenital lineages (T1, T2) (Table 2). We found classical ocular genotypes B/Ba and C segregating multiple times in the urogenital lineages, consistent with previous reports and once again underlining the danger of using genotyping as a proxy for phylogenetic relatedness (Supplemental Fig. S3). Importantly, some of these isolates, which have a urogenital backbone and ocular *ompA* genes, were associated both with urogenital infections and ocular disease in children up to 9 yr of age, a finding detailed by Andersson et al. (2016). We find European isolates that are almost identical, including *ompA* type, showing that these strains are not geographically isolated.

**Figure 5.**
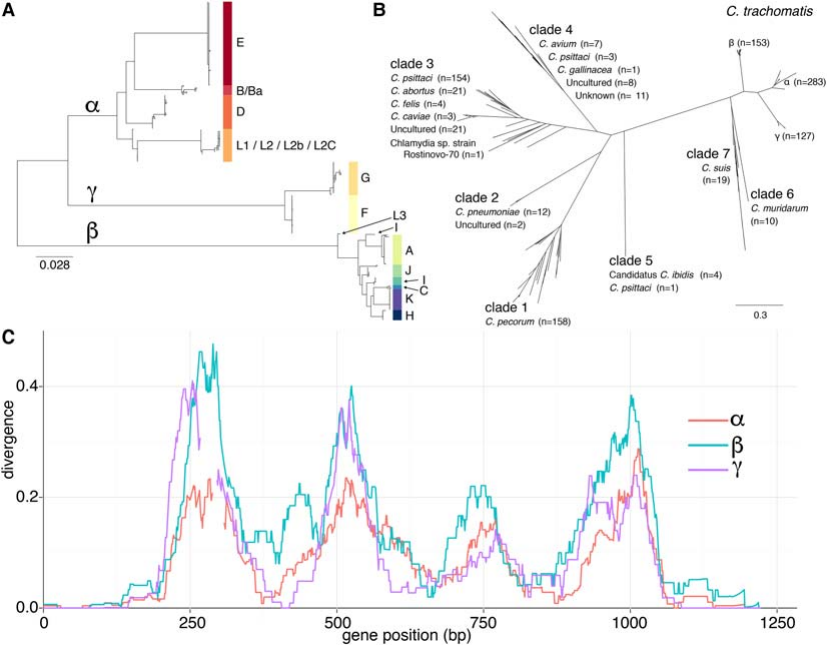
Diversity of the major outer membrane protein (MOMP) gene *ompA*. (*A*) Phylogenetic relationship between all 563 *Chlamydia trachomatis* isolates (*ompA* gene) shows separation into three clades labeled α, β, and γ. (*B*) Phylogenetic tree of 1003 MOMP-encoding genes across the *Chlamydiaceae*. (*C*) Divergence (proportion of differing nucleotides) of the *ompA* gene in three *C. trachomatis* clades compared with that of a reconstructed ancestor, shading indicates 10th and 90th percentiles.

The gene *ompA* is associated with phylogeny and tropism, despite experiencing the highest rates of observable recombination in the genome. The *C. trachomatis ompA* gene phylogeny reveals three clear lineages (labeled α, β, and γ) (Fig. 5A), which are broadly inconsistent with both tropism and whole-genome phylogeny. For instance, the “ocular” *ompA* types appear phylogenetically unrelated, while all LGV *ompA* types group together consistent with the whole genome. Four variable regions provide the majority of variation within the *ompA* gene with the regions at the 5′ and 3′ ends of the gene showing the most conservation (Fig. 5C), a pattern that was conserved across all members of the genus studied and may facilitate homologous recombination (Supplemental Fig. S6). Comparison between major outer membrane protein (MOMP)–encoding genes in other members of the *Chlamydiaceae* revealed evidence for between-species recombination in other members of the genus, but the *C. trachomatis ompA* forms a monophyly most closely related to those of *Chlamydia muridarum* and *Chlamydia suis* (Fig. 5B). The pattern of divergence from the reconstructed ancestor is not uniform among the clades, which may be due to differing selection pressures. Indeed, we found two regions in the *C. trachomatis ompA* clade β that are more closely related to ancestral *C. suis* isolates than other *C. trachomatis* clades (Supplemental Fig. S6).

### No evidence for antimicrobial resistance in circulating *C. trachomatis* populations

While chlamydial persistence is a reported but poorly understood phenomenon (Hogan et al. 2004), there is no evidence for stable antimicrobial resistance in a clinical setting (Sandoz and Rockey 2010). This is despite the ease at which mutations conferring a high level antimicrobial resistance to most clinically relevant antimicrobials can be generated in vitro (for review, see Sandoz and Rockey 2010) and sporadic reports of clinical resistance (Hogan et al. 2004; Sandoz and Rockey 2010), including a case of phenotypic but not genotypic resistance (O'Neill et al. 2013). Although none of the isolates in this study were deemed clinically resistant, in order to understand whether any of these mutations were present in a circulating population, we performed a systematic search for known resistance alleles, either fixed or heterozygous. We found absolutely no evidence for genomic resistance in this large, clinically relevant data set. This analysis included the 23S rRNA gene, which contains previously described resistance mutations to the macrolide azithromycin, the current frontline antimicrobial treatment. It is important to note that these samples were collected from patients for routine diagnosis prior to antibiotic treatment and were not from treatment failures.

## Discussion

It is unlikely that older or more diverse strains will ever be available for analysis on the scale presented here. Despite the geographical clustering present in the data, our sampled locations are spread across the phylogeny, giving us the necessary platform to draw broad conclusions about the current genomic picture of this pathogen, such that observations made here about the evolution of these lineages are likely to be applicable to the species as a whole.

Our data indicate two distinct phases in the evolution of *C. trachomatis*: deep variation and contemporary mixing. The phylogeny clearly shows four deeply divergent lineages that we estimate to have expanded over the last few thousand years, with the flatter appearance of lineage T1 potentially a signature of a more recent history of clonal expansions than found in T2 (Grenfell 2004). This overall structure is consistent with population bottlenecks such as isolation due to barriers created by geographical isolation facilitated by low host population density and lack of migration, which create barriers to recombination flow between groups (Fraser et al. 2007). We speculate that these populations would diverge, but themselves remain coherent through periodic selection by recombination and selective sweeps within the ecotype (Cohan 2001; Bendall et al. 2016). There is no evidence or requirement under this model for these lineages to be the result of introgression from other hosts; however, broader sampling, including of different host species, is required to truly clarify this hypothesis. Our analyses are not able to address the possibility of recombination between extant and unsampled lineages.

Contemporarily, we see large amounts of mixing between and within lineages. The lower rate of recombination between LGV and other lineages could be explained by a lower rate of coinfection, and therefore opportunity to recombine, with isolates from other lineages. Detectable recombination in this pathogen requires coinfection between diverse strains, and we hypothesize that this has become more frequent due to increases in human population density and mixing. We speculate that over time these recombinations will break down the deep phylogenetic structure we currently observe. While we see recent small-scale expansions in the phylogeny that correlate with source location and genotype, we expect each of these to be transient in nature and, in time, to disappear as these clades expand and are subject to recombination with strains outside their niche.

There are three notable exceptions to these trends. The epidemiologically driven expansion of the L2b monophyly is most probably due to their epidemiological association to the population of men who have sex with men. Thus, this expansion is a direct result of a niche created by human behavior, as observed in other pathogens such as *Shigella flexneri* serotype 3a (Baker et al. 2015). Second, ocular disease is today mostly understood in the context of the ocular lineage, which has maintained genetic coherence potentially due to geographical factors. Recent results (Andersson et al. 2016), included here, indicate that the trachoma lineage is not the only source for ocular disease and that this disease may be more ancient and genetically diverse than the ocular lineage alone. Finally, the expansion of genotype E, currently the most prevalent genotype, may be due to increased fitness at or around *ompA*, preventing recombinations being fixed in this region, being simply a stochastic increase, or being a combination of the two. These exceptions are fascinating and reinforce the fact that the evolutionary dynamics of *C. trachomatis* are intertwined with human behavior and population structure.

We noted across all our metrics that many of the variable regions are focused around membrane proteins (*omp*, *pmp, inc*), which are believed to be targets of immune response. We know that different lineages often inhabit different niches, and we have for the first time identified the differing signatures between lineages that are most likely due to changing tropic or immune pressures across the species. These results provide an invaluable resource for prospective in vitro studies or for the selection of appropriate vaccine candidates.

At a time in which nearly every human pathogen is becoming resistant to antimicrobials, *C. trachomatis* stands apart. It is interesting to contrast this with *N. gonorrhoeae*, with which it frequently coinfects, which is becoming a global health concern due to acquired genetic resistance. Our finding that there is absolutely no genetic evidence of resistance in circulating *C. trachomatis* is surprising given that resistance can easily evolve in vitro and that sporadic treatment failures do occur. While the sample collection did not include treatment failures, prior exposure to antibiotics (for any reason) of patients who visited an STI clinic for either *C. trachomatis* or *N. gonorrhoeae* has been reported to be ∼10% (Dukers-Muijrers et al. 2014). Thus, although these findings do not preclude the evolution of resistance in clinical cases, they show that any such mutations are not in the circulating *C. trachomatis* clinical population sampled in this study.

If reported treatment failures are a result of genetic mutations, these have not spread, implying that the fitness burden of resistance is too high for successful transmission. The rapid rise of the Swedish new variant of *C. trachomatis* (nvCt) (Seth-Smith et al. 2009; Unemo et al. 2010; Unemo and Clarke 2011), in which a deletion in the plasmid allowed diagnostic escape, shows that minority variants can rise in frequency to become fixed within a host and that given the right selective conditions, such as treatment escape, variants can quickly spread throughout the population. Given that this pathogen has been one of the two most common bacterial STIs during the entire antimicrobial era, the likelihood of resistance emerging and spreading now seems low.

## Methods

### *C. trachomatis* strains and DNA extraction

Samples (*n* = 563) were collected from 21 countries and include historical and current samples spanning over 50 yr (1957–2012). Previously published sequences, as well as resequenced strains, were also included in the analysis. DNA for sequencing was isolated directly from clinical swabs, from cultured collections, as well as from historical isolates propagated in yolk sac of embryonated hens’ eggs. DNA was processed using a variety of methods, including IMS (using an antibody against *C. trachomatis* lipopolysaccharide) with or without MDA (Seth-Smith et al. 2013a), as well as a custom SureSelect enrichment system similar to that recently described by Christiansen et al. (2014) that allows sequencing from clinical swabs stored in lysis buffer. Details of all strains and methods used are in Supplemental Table S1.

### Sequencing, mapping, and quality control

Sequencing was performed using Illumina HiSeq with multiplexing using paired read lengths of between 75 and 100 bp. In order to check coverage and quality, sequences were first mapped to a published reference chromosome specific to their lineage (T1: F/SW4, T2: D/UW3, LGV: L2/434, ocular: A/HAR-13) (for accession numbers, see Supplemental Table S1) using SMALT (version 0.7.4, 90% minimum read identity; www.sanger.ac.uk/resources/software/smalt/) and GATK insertion/deletion realignment (McKenna et al. 2010). Sequences with less than 5× mean coverage across 95% of the chromosome were excluded, as were sequences with uneven coverage, indicated by a coefficient of variation greater than one (Seth-Smith et al. 2013b). SNPs were called using previously described methods (Harris et al. 2012), and short insertions/deletions were included in the resulting alignment. To identify samples with mixed *C. trachomatis* populations, we identified heterozygous sequences by identifying all positions with minor allele frequencies >0.2 as determined by BCFtools v0.1.19 (Li et al. 2009; http://samtools.github.io/bcftools/) and excluding those sequences with more than 300 such positions (mean 105 bp; standard deviation 600 bp). For consistent analysis, we created a pseudogenome to represent all the diversity observed in a previously published species-wide analysis (Harris et al. 2012) with gene names as per the published D/UW3 strain (Stephens et al. 1998), to which all samples were remapped.

### Genotyping

We genotyped samples by mapping reads to a panel of 28 reference *ompA* sequences chosen to represent all previously published genotypes. Genotypes B/Ba, I/Ia, and J/Ja are grouped together since the *ompA* sequence differences between these pairs of genotypes are minimal. Samples were mapped against this panel using the same parameters given in the previous section. Genotypes were allocated by minimizing pairwise differences. Where multiple references had fewer than two SNPs against the samples, the results were manually checked using Artemis (Carver et al. 2012) to ensure accurate mapping.

### Phylogenetics and recombination detection

Initial chromosome and plasmid phylogenies were constructed using RAxML (Stamatakis et al. 2005) from a variable sites alignment using *C. muridarum* as an outgroup (this outgroup is not displayed in figures). Putative recombination regions were identified as previously published (Croucher et al. 2011). In brief, regions of increased SNP density relative to the phylogenetic neighbors were identified and removed in an iterative fashion. Final phylogenies were constructed using RAxML on the resulting (recombination removed) alignment.

Pairwise sharing of traits was investigated by calculating the proportion of pairs of isolates below a certain distance (patristic distance from the recombination removed tree) that share the same trait. One hundred permutations of the traits were computed to test the null hypothesis that the trait was not correlated with difference and to calculate the statistical significance of any observed difference.

### Recombination analysis

To investigate the genes affected by recombination and the differences between lineages, we scored each gene with the number of recombination events overlapping that gene in each lineage. To correct for any sampling differences, scores in each lineage were represented by the number of standard deviations from the mean (of the lineage). *k*-means clustering was used to partition all genes into groups with the number of groups chosen by inspecting the sum of squared error plot. To explore whether recombination was due to donors from the same lineage, we classed homoplasic SNPs as those confined to a certain lineage as opposed to those found in multiple lineages. The number of such positions were scaled by the synapomorphic SNPs in a 10-kb sliding window at 1-kb intervals across the genome, similar to the method previously described (Everitt et al. 2014).

### Dating analysis

Only samples with associated sample collection dates were included in this analysis with putative recombination events removed from the genomic data. Root-to-tip regression analysis was used to date the phylogeny. The significance of this regression was estimated by comparison of the *R*^2^ values between 100 permutations. BEAST version 1.8.0 (Drummond and Rambaut 2007) was run in triplicate under a strict clock and a number of population models (constant population size, exponential growth, and Bayesian skyline with four groups). In all three cases, the triplicates converged (effective sample size > 200 and highly similar parameter estimate distributions), and the inferred tree height and mutation rates were comparable.

### MOMP comparisons

In addition to the *C. trachomatis ompA* sequences, 440 genes >500 bp were extracted from NCBI via the search term (“Chlamydia/Chlamydophila group”[Organism] NOT “Chlamydia trachomatis”[Organism]) AND (*ompA*[Gene Name] OR MOMP[Gene Name]). MUSCLE version 3.8.31 (Edgar 2004) was used to align these genes, and a phylogenetic tree was drawn as before. Pairwise differences were calculated by comparison with a given reference and used a 50-bp sliding window with 1-bp step size. FastML (Pupko et al. 2000) was used to reconstruct the *C. trachomatis* ancestor.

### Putative resistance mutations and 23S rRNA mapping

The high depth of sequence coverage obtained allows identification of allele variants that are present but not fixed (as SNPs) in samples. Short-read mapping data were investigated at positions that confer antimicrobial resistance in vitro in the literature. Heterozygous alleles were identified as positions with multiple alleles each with a per-strand read depth above five where mapping and base (phred) quality was above 30, and optical duplicates were removed. As the reference *C. trachomatis* genome has two (identical) copies of the rRNA operon, one copy of the 23S rRNA gene was masked out during mapping. Kraken (Wood and Salzberg 2014) analysis of reads mapping to this region indicated that a proportion were from other species. We therefore employed differential mapping to one copy of the *C. trachomatis* rRNA operon and the corresponding operon from *Lactobacillus salivarius* strain UCC118 (the highest species match from multiple Kraken results) and additionally required that mapped reads were properly paired (i.e., both reads map to the operon). Differential mapping is essential as DNA from other species is often extracted and subsequently sequenced at low depth. Due to the high similarity of rRNA regions, this may cause the appearance of heterozygous SNPs and possible resistance alleles (Supplemental Fig. S7).

## Data access

The sequencing data from this study have been submitted to the European Nucleotide Archive (ENA; http://www.ebi.ac.uk/ena) under the accession numbers listed in Supplemental Table S1.

## Supplementary Material

Supplemental Material
