# Supplementary material for: Comprehensive global genome dynamics of Chlamydia trachomatis show ancient diversification followed by contemporary mixing and recent lineage expansion
Source: Genome Res. 2017 Jul;27(7):1220–9. doi: 10.1101/gr.212647.116 (PMC5495073; doi:10.1101/gr.212647.116)
Supplement: Supplemental Material [file supp_27_7_1220__index.html]

Comprehensive global genome dynamics of Chlamydia trachomatis show ancient diversification followed by contemporary mixing and recent lineage expansion — Supplemental Material 

# Comprehensive global genome dynamics of *Chlamydia trachomatis* show ancient diversification followed by contemporary mixing and recent lineage expansion

## Supplemental Material

- Supplemental\_Table\_S3.xlsx
- Supplemental\_Fig\_S7.pdf
- Supplemental\_Table\_S1.xlsx
- Supplemental\_Table\_S2.xlsx
- Supplemental\_Fig\_S1.pdf
- Supplemental\_Fig\_S4.pdf
- Supplemental\_Fig\_S2.pdf
- Supplemental\_Fig\_S5.pdf
- Supplemental\_Fig\_S3.pdf
- Supplemental\_Fig\_S6.pdf
