# Supplementary material for: Comprehensive global genome dynamics of Chlamydia trachomatis show ancient diversification followed by contemporary mixing and recent lineage expansion
Source: Genome Res. 2017 Jul;27(7):1220–9. doi: 10.1101/gr.212647.116 (PMC5495073; doi:10.1101/gr.212647.116)
Supplement: Supplemental Material [file supp_gr.212647.116_Supplemental_Fig_S7.pdf]

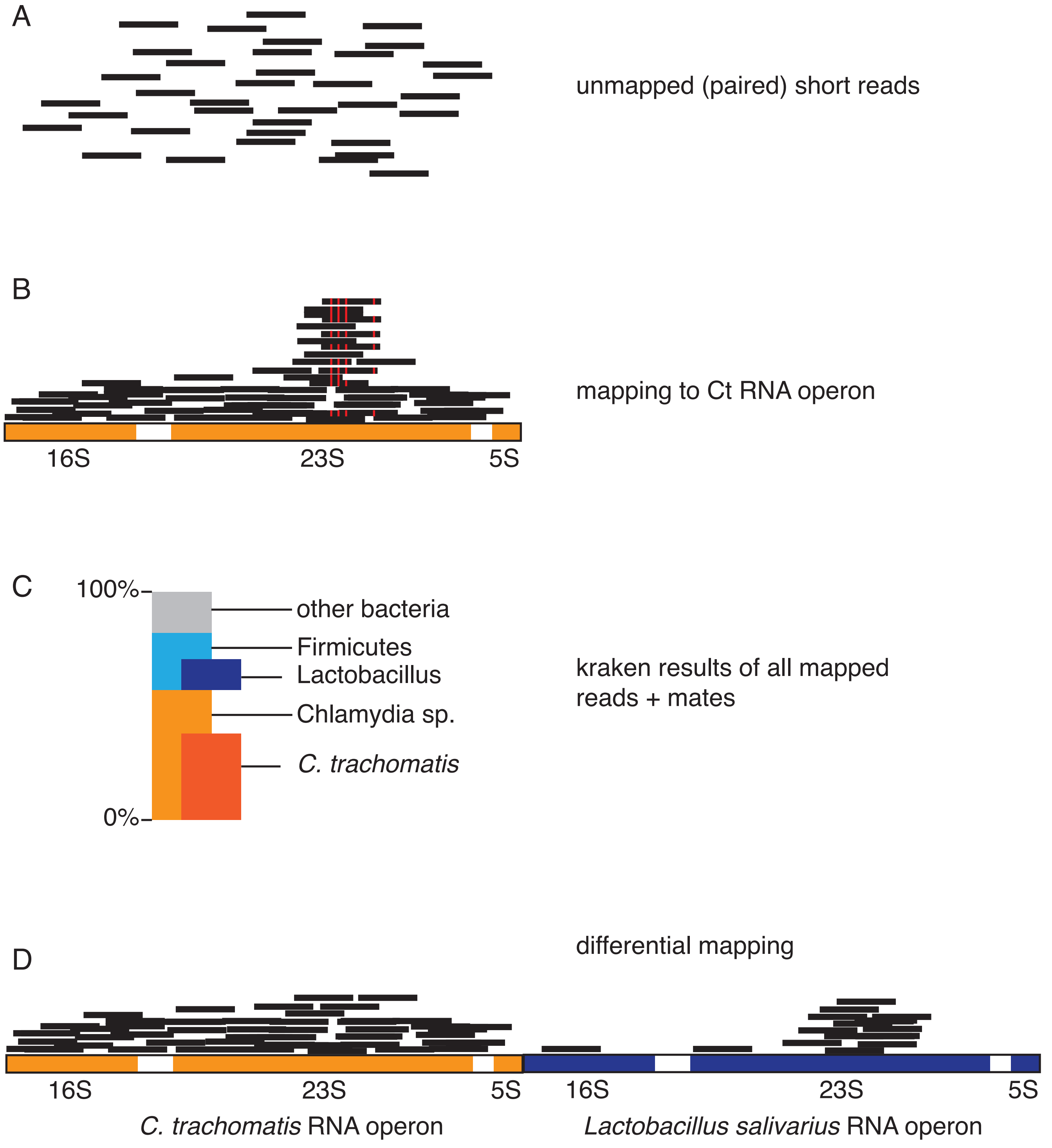


Supplemental Fig S7 A workflow for reducing the impact of contamination from foreign DNA. Short read data (A) is mapped to the reference operon/genome of interest (B). Kraken analysis (C) reveals that a high proportion of the mapped reads are not from the species of interest. When the reads are differentially mapped to the operon from both species (D) the mapping quality is markedly improved, as evident from the reduction in polymorphisms and the more even coverage.
