# Supplementary material for: Comprehensive global genome dynamics of Chlamydia trachomatis show ancient diversification followed by contemporary mixing and recent lineage expansion
Source: Genome Res. 2017 Jul;27(7):1220–9. doi: 10.1101/gr.212647.116 (PMC5495073; doi:10.1101/gr.212647.116)
Supplement: Supplemental Material [file supp_gr.212647.116_Supplemental_Fig_S1.pdf]

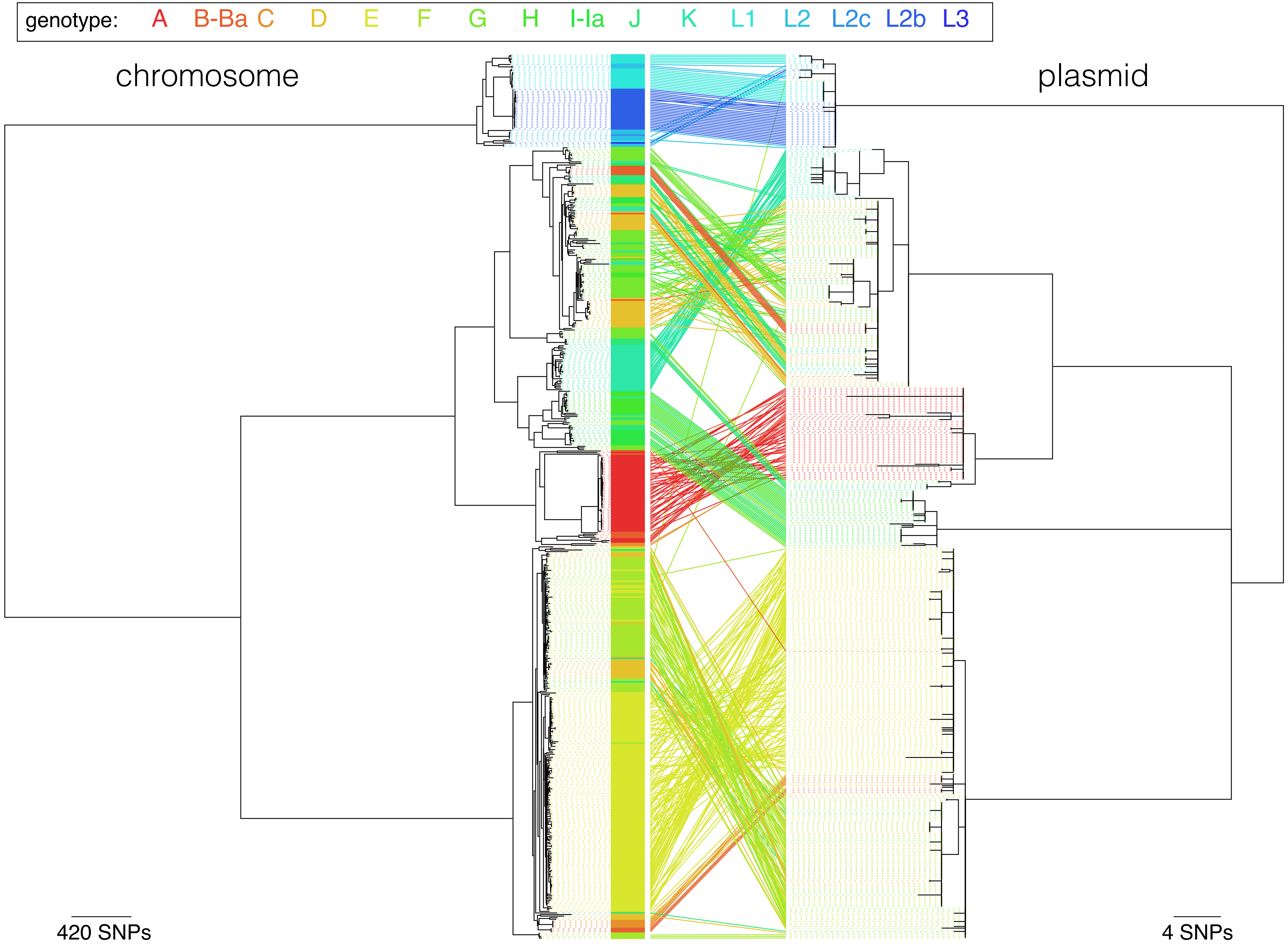

Supplemental Fig S1 Comparison of genome (left) and plasmid (right) phylogenies with tips matched. The incongruence of the phylogenies indicates that inheritance is not always vertical.
