# Supplementary material for: Comprehensive global genome dynamics of Chlamydia trachomatis show ancient diversification followed by contemporary mixing and recent lineage expansion
Source: Genome Res. 2017 Jul;27(7):1220–9. doi: 10.1101/gr.212647.116 (PMC5495073; doi:10.1101/gr.212647.116)
Supplement: Supplemental Material [file supp_gr.212647.116_Supplemental_Fig_S4.pdf]

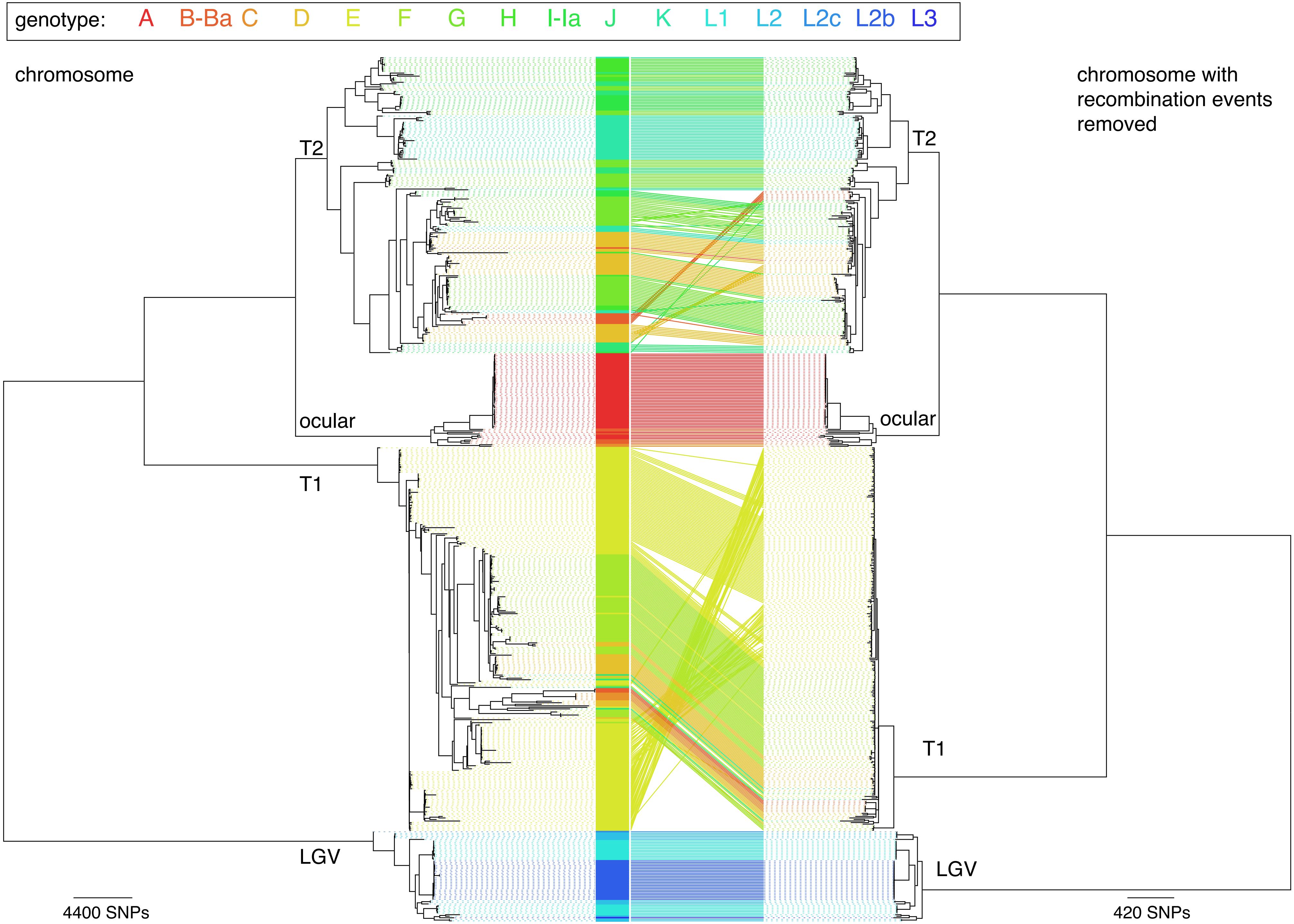

Supplemental Fig S4 Tanglegram of maximum-likelihood trees between chromosome (left) and chromosome with recombinations removed (right). Scale bars show single nucleotide polymorphism (SNP) differences and are specific to each tree.
