# Supplementary material for: Comprehensive global genome dynamics of Chlamydia trachomatis show ancient diversification followed by contemporary mixing and recent lineage expansion
Source: Genome Res. 2017 Jul;27(7):1220–9. doi: 10.1101/gr.212647.116 (PMC5495073; doi:10.1101/gr.212647.116)
Supplement: Supplemental Material [file supp_gr.212647.116_Supplemental_Fig_S2.pdf]

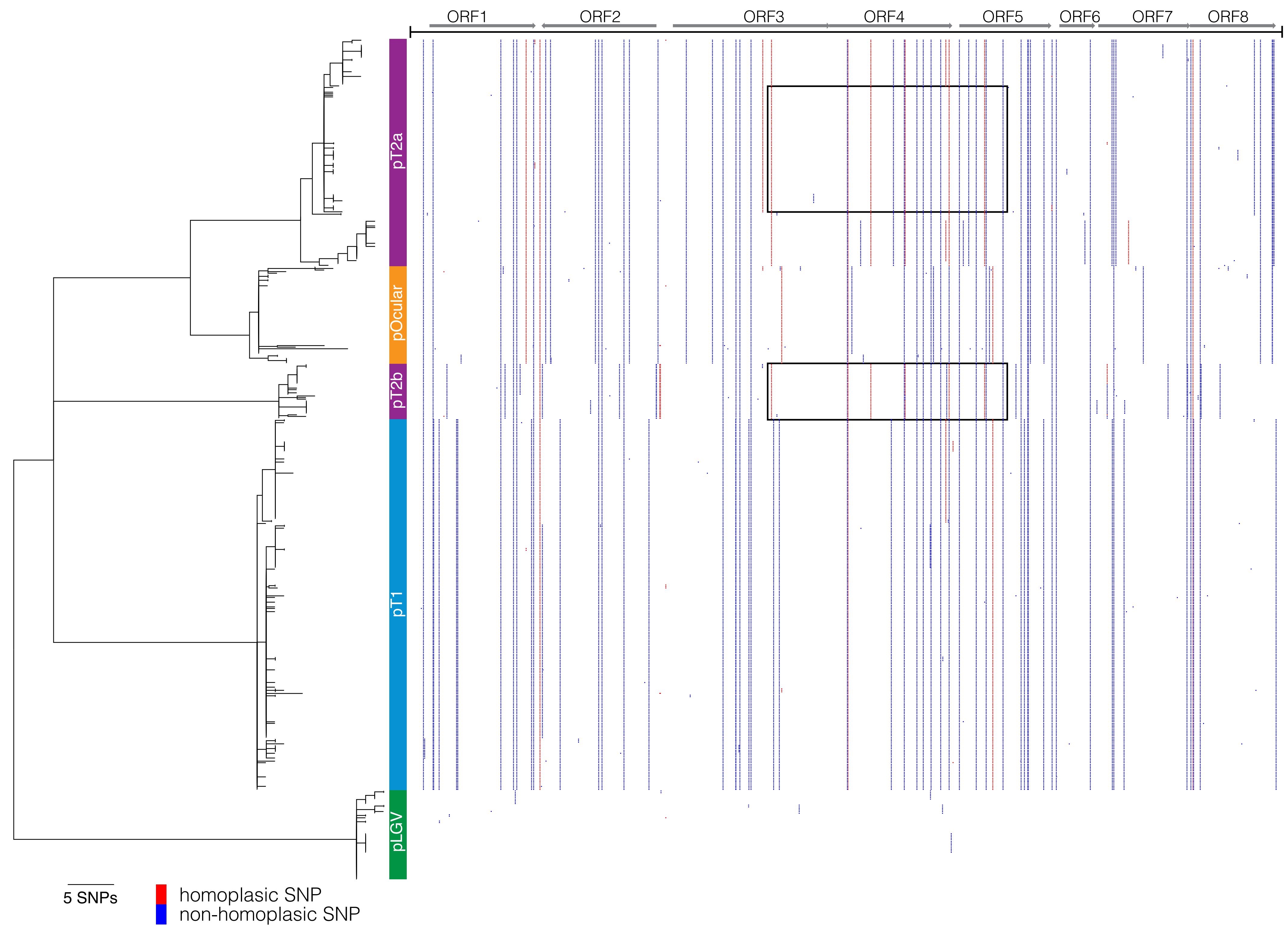

Supplemental Fig S2 Plasmid phylogeny with single nucleotide polymorphisms (SNPs) (red: homoplasic SNPs) corresponding to an annotated and linearized plasmid. Identified block corresponds to a recombination or shared ancestry event between two clades (pT2a, pT2b) which are contained in T2 isolates.
