# Supplementary material for: Comprehensive global genome dynamics of Chlamydia trachomatis show ancient diversification followed by contemporary mixing and recent lineage expansion
Source: Genome Res. 2017 Jul;27(7):1220–9. doi: 10.1101/gr.212647.116 (PMC5495073; doi:10.1101/gr.212647.116)
Supplement: Supplemental Material [file supp_gr.212647.116_Supplemental_Fig_S5.pdf]

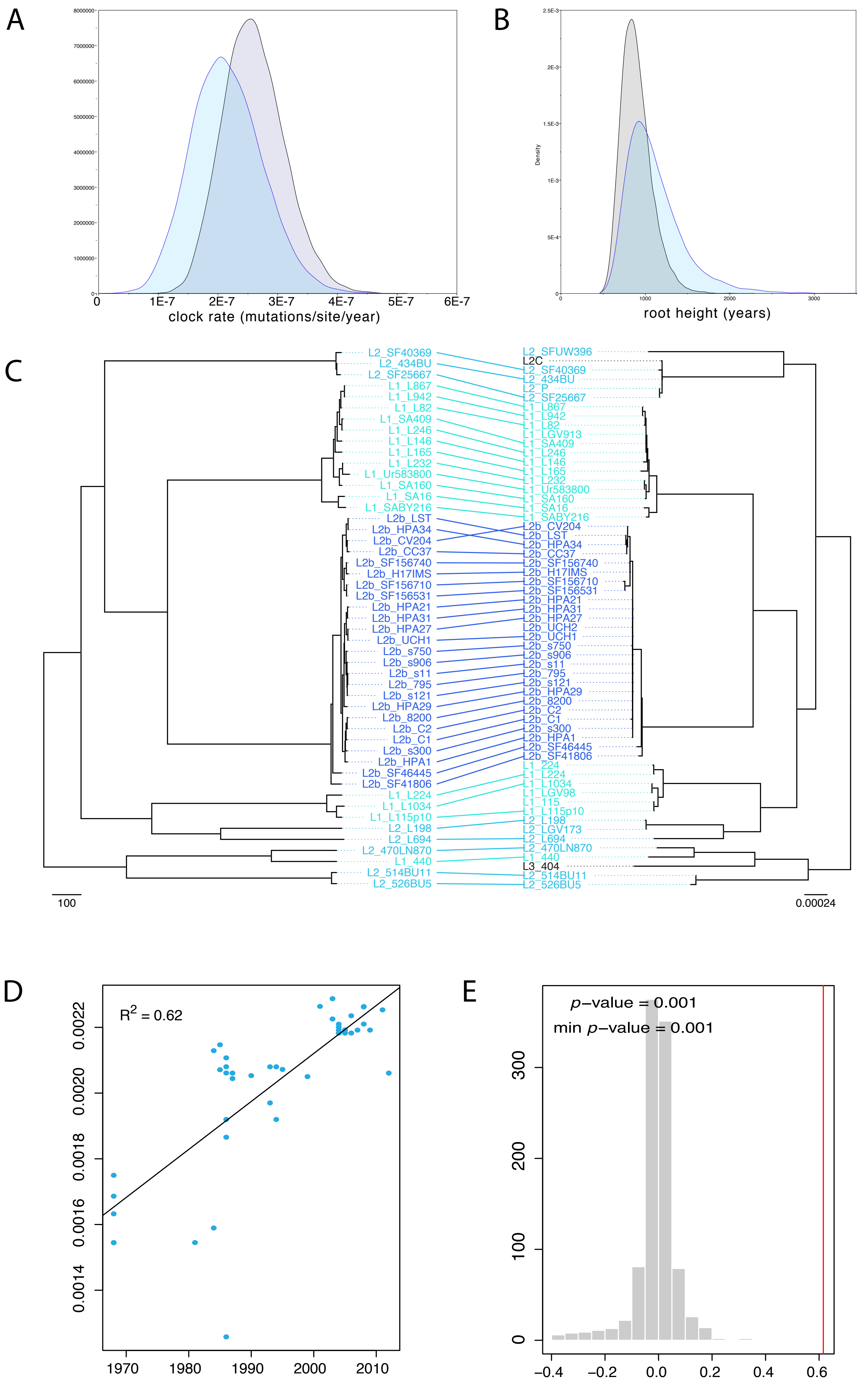

Supplemental Fig S5 Comparison of estimated molecular clock rate (A) and tree root height (B) between two different population models, exponential growth and Bayesian skyline. Tree topology is extremely similar between Bayesian and Maximum Likelihood methods (C), taxa not present in the Bayesian construction did not have associated collection dates. Linear regression of root-to-tip distance and collection date shows a strong temporal signature (D) which is not maintained when the dates are permuted (E).
