# Supplementary material for: Comprehensive global genome dynamics of Chlamydia trachomatis show ancient diversification followed by contemporary mixing and recent lineage expansion
Source: Genome Res. 2017 Jul;27(7):1220–9. doi: 10.1101/gr.212647.116 (PMC5495073; doi:10.1101/gr.212647.116)
Supplement: Supplemental Material [file supp_gr.212647.116_Supplemental_Fig_S3.pdf]

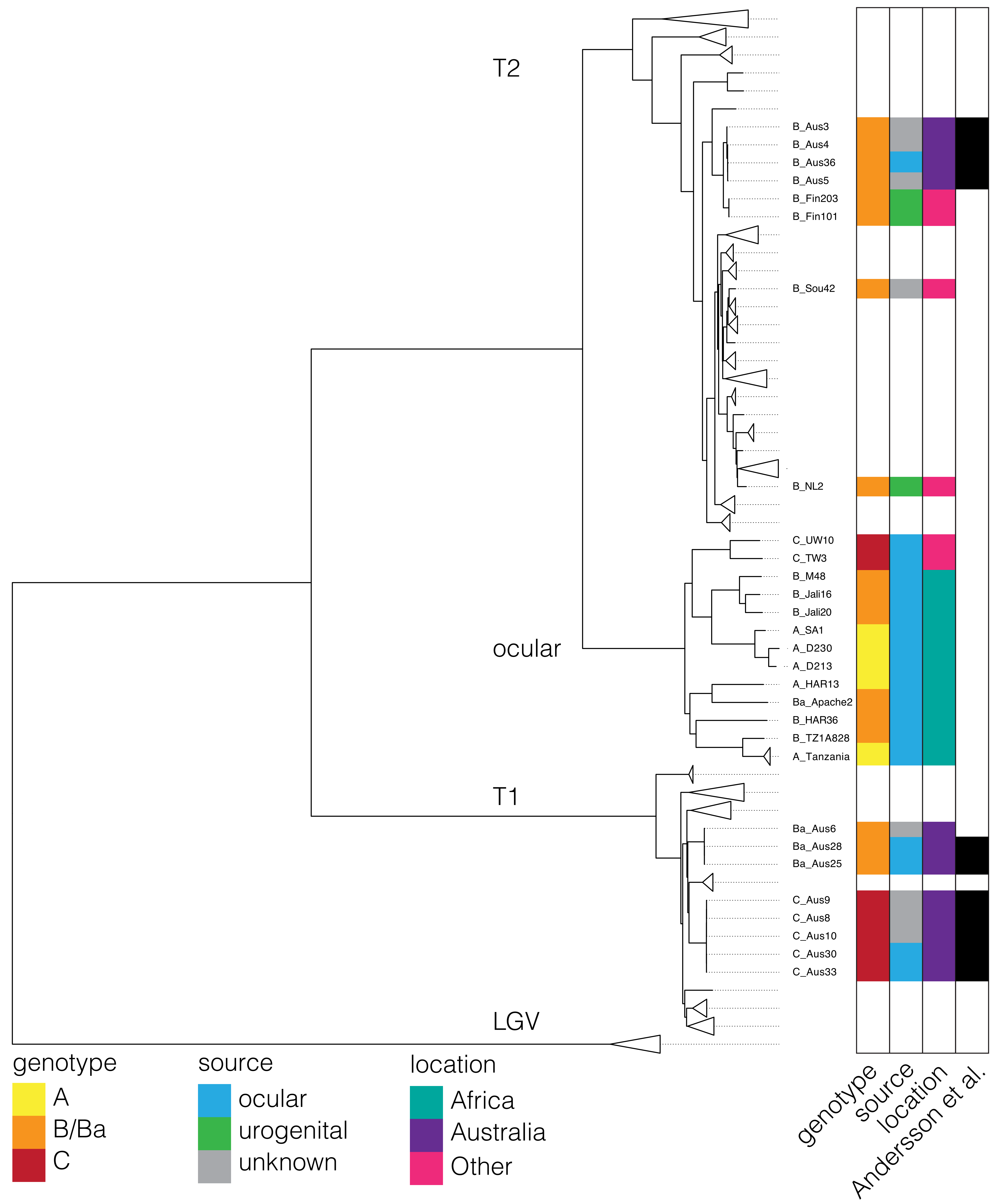

Supplemental Fig S3 Collapsed species phylogeny highlighting the segregation of the B/Ba and C genotypes. There have been multiple introductions of ocular genotypes into both "urogenital" lineages and these introductions have been found in both ocular and urogenital infections.
