# Supplementary material for: Comprehensive global genome dynamics of Chlamydia trachomatis show ancient diversification followed by contemporary mixing and recent lineage expansion
Source: Genome Res. 2017 Jul;27(7):1220–9. doi: 10.1101/gr.212647.116 (PMC5495073; doi:10.1101/gr.212647.116)
Supplement: Supplemental Material [file supp_gr.212647.116_Supplemental_Fig_S6.pdf]

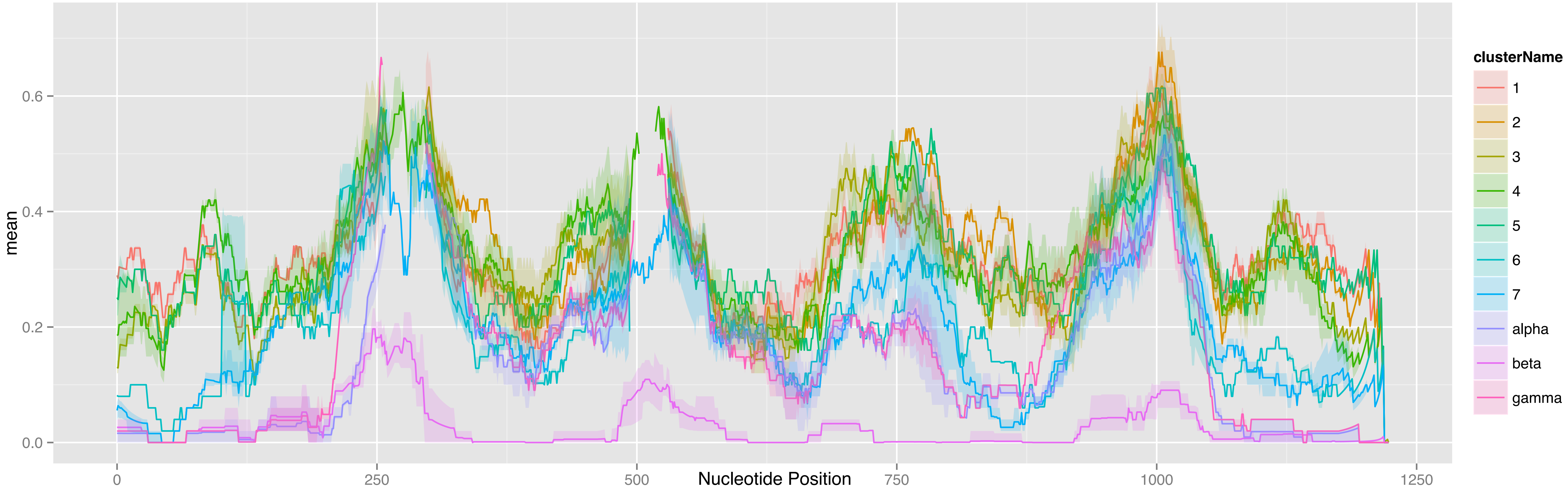

Supplemental Fig S6 Pairwise comparison of major outer membrane protein (MOMP) clades found in Figure 5C with L1_404 (the most ancestral Chlamydia trachomatis isolate obtained in 1967). Shading shows 10th and 90th percentile.
